# Quantitative G6PD Deficiency Screening in Routine Malaria Diagnostic Units in the Brazilian Amazon (SAFEPRIM): An Operational Mixed-Methods Study

**DOI:** 10.3390/pathogens11111328

**Published:** 2022-11-11

**Authors:** Jose Diego Brito-Sousa, Felipe Murta, Sheila Vitor-Silva, Vanderson Sampaio, Maxwell Mendes, Brenda Souza, Talita Batista, Alicia Santos, Leonardo Marques, Laila Barbosa, Patricia Balieiro, Alexandre Silva-Neto, Renata Rabello, Marcelo Brito, Emanuelle Silva, Sheila Rodovalho, Ana Ruth Arcanjo, Gisely Melo, Judith Recht, Gonzalo J. Domingo, Suiane Valle, Rodrigo Souza, Theresa Nakagawa, Wuelton Monteiro, Marcus Lacerda

**Affiliations:** 1 Fundação de Medicina Tropical Dr Heitor Vieira Dourado, Instituto de Pesquisa Clínica Carlos Borborema, Manaus 69040-000, Brazil; 2 Escola Superior de Ciências da Saúde, Universidade do Estado do Amazonas, Manaus 69065-001, Brazil; 3 Escola de Enfermagem de Manaus, Universidade Federal do Amazonas, Manaus 69057-070, Brazil; 4 Fundação de Vigilância em Saúde Dra Rosemary Costa Pinto—FVS-RCP, Manaus 69093-018, Brazil; 5 Pan American Health Organization—PAHO, World Health Organization, Brasilia 70312-970, Brazil; 6 Laboratório Central de Saúde Pública do Amazonas—LACEN/AM, Manaus 69020-040, Brazil; 7 Independent Consultant, North Bethesda, MD 20852, USA; 8 Diagnostics Program, PATH, Seattle, WA 98121, USA; 9 Hospital Regional do Juruá, Cruzeiro do Sul 69895-000, Brazil; 10 Campus Floresta, Universidade Federal do Acre, Cruzeiro do Sul 69895-000, Brazil; 11 Fundação Oswaldo Cruz, Brasilia 70904-130, Brazil; 12 Fundação Oswaldo Cruz, Instituto Leônidas e Maria Deane—ILMD, Manaus 69057-070, Brazil

**Keywords:** G6PD deficiency, *Plasmodium vivax*, implementation, malaria, elimination, primaquine, operational study, Standard G6PD, Brazil, SD biosensor, quantitative G6PD test

## Abstract

Background: Glucose-6-phosphate dehydrogenase (G6PD) deficiency testing is not routinely performed before primaquine treatment in most *Plasmodium vivax* endemic areas, despite the risk of primaquine-associated hemolysis. This is due to the operational challenges associated with pragmatic G6PD testing and as such needs to be addressed. Methods and findings: This mixed-methods operational study was aimed at implementing the quantitative point-of-care Standard^TM^ G6PD (SD Biosensor, Korea) screening test in malaria treatment units (MTUs) in the municipalities of Rio Preto da Eva and Mâncio Lima, in the Brazilian Amazon, between mid-January 2020 and December 2020. In total, 1286 *P. vivax* cases were treated based on the Standard G6PD test: 1230 had activity equal to or greater than 4.0 U/g Hb, and 56 less than 4.0 U/g Hb. No G6PD deficient (G6PDd) genotypes were found in 96 samples from the 1230, and only 21 of the 56 G6PDd cases had confirmed G6PDd genotypes. Evaluations were conducted on the proficiency of health care professionals (HCPs) training to perform the test, the reliability of testing performed in the field, and the perceptions of HCPs and patients about the implementation. Post-training proficiency was 73.4% after a 4-hour training session. This study revealed that locations with lower malaria caseloads will need regular refresher training. The test was well accepted by both HCPs and patients. Signs and symptoms of hemolysis were not always associated with malaria treatment drugs by HCPs and patients. Interpretation: Point-of-care quantitative G6PD testing can be performed at MTUs in the Brazilian Amazon to inform treatment decisions with primaquine. Limitations related to technical and cultural aspects need to be addressed further when expanding screening to larger areas.

## 1. Introduction

The use of primaquine (PQ), and the newly-approved tafenoquine (TQ), for the radical cure of *Plasmodium vivax* can lead to acute hemolytic anemia (AHA) in patients presenting with glucose-6-phosphate dehydrogenase deficiency (G6PDd) [1]. Red blood cells are susceptible to early destruction due to oxidative stress triggered by 8-aminoquinolines, an active ingredient of PQ [2,3]. In Brazil, G6PDd affects around 5% of the population in malaria endemic areas and the resulting AHA can lead to potentially fatal severe anemia and acute kidney injury [4,5,6]. Nonetheless, G6PDd screening is not routinely performed on vivax malaria patients prior to treatment in Brazil, where all patients receive a standard 7-day PQ treatment (0.5mg/kg/day) [7]. As a result, G6PDd is often only diagnosed as the condition worsens after the onset of hemolysis [4,5,8].

The qualitative CareStart^TM^ screening test was previously evaluated in Rio Preto da Eva in Brazil, proving highly sensitive but with low specificity, leading to a significant portion of individuals missing out on the radical cure [9]. Limitations of qualitative testing platforms can be overcome by the availability of quantitative point-of-care tests for G6PDd, especially in diagnosing heterozygous females with intermediate G6PD activity who remain at risk of hemolysis and are not always detected in qualitative tests [10,11,12]. Quantitative tests, however, require different skills and abilities to conduct the tests and interpret results. 

Thus, the present study aimed at implementing the routine use of the Standard™ G6PD quantitative test at malaria treatment units (MTUs) in two municipalities in the countryside of the Brazilian Amazon, assessing the reliability of interpretation by health care professionals (HCPs), the test performance in the field, the clinical impact of test implementation to prevent/avoid AHA cases after malaria diagnosis, and the perceptions of HCPs and patients about the test implementation.

## 2. Methods

### 2.1. Study Design

The SAFEPRIM mixed-methods study evaluated the operational aspects of implementing the Standard^TM^ G6PD (SD biosensor, Korea) quantitative test for G6PDd screening prior to PQ use in the Brazilian public health system (SUS). The effectiveness of the training, reliability of the test performed in the field, and the perceptions of HCPs and patients about the test implementation were analyzed within the scope of this implementation. Specific details of each aspect analyzed are described below.

### 2.2. Study Sites

**Figure 1:**
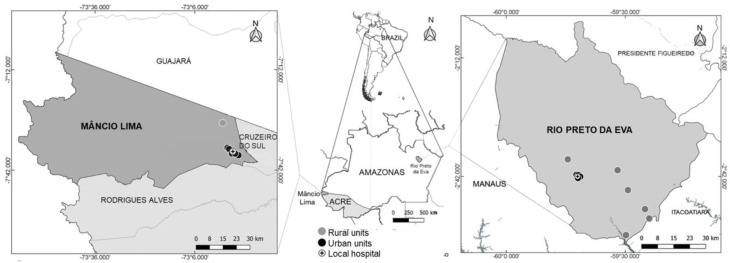
**Study locations and malaria treatment units (MTU).** Rio Preto da Eva is located 78 km from Manaus, the capital of the state of Amazonas. Mâncio Lima is in the Jurua Valley region, 617 km away from the capital city of the state of Acre, Rio Branco. The test was implemented in: urban units, including the local hospital, black circles and black cross, respectively, and rural health posts (grey circles). In total, 18 units were included in the study (7 in Mâncio Lima and 11 in Rio Preto da Eva) plus extra rotating devices for active surveillance visits. The nearest reference institutions are the *Fundação de Medicina Tropical Dr Heitor Vieira Dourado* (Manaus, state of Amazonas) and the *Hospital do Juruá* (Cruzeiro do Sul, state of Acre). This map was created based on the Brazilian Institute of Geography and Statistics (https://portaldemapas.ibge.gov.br/portal.php#homepage accessed on 23 July 2021).

The quantitative G6PD screening test was implemented in 18 MTUs in urban and rural areas, including local hospitals, within the Brazilian municipalities of Rio Preto da Eva (early January to August) and Mâncio Lima (January to December) in 2020 (Figure 1), pursuant to an agreement signed by the local, state, and federal malaria control programs. Mâncio Lima is the western-most city in Brazil, located in the Jurua Valley region, with an estimated population of 19,311 inhabitants, 617 km away from Rio Branco, the capital city of Acre. The nearest specialized health facility is in Cruzeiro do Sul, approximately 30 km by road. Rio Preto da Eva is located 78 km away from the capital city Manaus by road in the state of Amazonas, with an estimated population of 32,577 inhabitants. Some distant health units were not included in the implementation in Mâncio Lima due to their challenging locations for clinical management and supervision. Mâncio Lima had a higher annual parasite index (API) (91.8) compared to Rio Preto da Eva (17.2) in 2020 [13].

### 2.3. HCPs Training and Proficiency Assessment

Training and evaluation of proficiency were coordinated with local and state reference institutions to ensure sustainability of the actions post-study. Community health workers and malaria control workers from all units were trained at locations provided by each municipal Secretariat of Health. Job aids and training structure were adapted from the implementation of another G6PD screening test (CareStart^TM^, AccessBio, USA) in Rio Preto da Eva [9]. Theoretical and practical training sessions lasting approximately 4 h covered G6PDd testing, malaria treatment, pharmacovigilance of PQ-associated hemolysis, Standard^TM^ G6PD test procedure, interpretation, result documentation, and patient counselling. Job aids included a locally recorded video of the Standard^TM^ G6PD procedure, a guideline folder summarizing the test procedure and interpretation, a guidance poster for every MTU, and a modified (*Sistema de Informação de Vigilância Epidemiológica;* Epidemiological Surveillance Information System) SIVEP-malaria reporting form adapted from the nationally used version to add G6PD data and pharmacovigilance of PQ-associated hemolysis. All training materials are publicly available from the G6PD Operational Research Community of Practice [14]. 

A supervised practice session followed the theory session, where groups of five to eight HCPs per instructor could practice all steps. A post-training theory and practice examination (Supplementary file S1) required 90% (or no more than one wrong answer) as a satisfactory result, with additional individual training and evaluation in case of a failed attempt. Training certificates were provided after approval in both tests. Proficiency assessments to check knowledge consolidation were carried out at six, and at six and twelve months post-implementation in Rio Preto da Eva and Mâncio Lima, respectively. The training materials were distributed as refreshers. Assessments were carried out using controls provided by the manufacturer.

### 2.4. G6PD Testing, Results Recording and Pharmacovigilance of Hemolysis

The Standard^TM^ G6PD test measures the concentration of total hemoglobin (g/dL) and G6PD enzymatic activity (IU/g Hb) in fresh human whole blood based on reflectometry assays [11,15]. Briefly, a device/strip is inserted into the analyzer. Using the SD Ezi tube+ sample collector, 10 µL of capillary blood is transferred to the extraction buffer tube, followed by thorough mixing with the SD Ezi tube+ sample collector. A new SD Ezi tube+ sample collector is used to draw a 10 µL mixed specimen and extraction buffer to apply to the test device. After 2 min, the hemoglobin measurement in g/dL and the quantitative ratio of G6PD to hemoglobin (IU/g Hb) are shown simultaneously. The Standard^TM^ G6PD test is designed to function over an operating temperature range of 15–40 °C and has a dynamic range for specific G6PD activity of 0–20 IU/g Hb. The cut-off for deficiency suggested by the manufacturer is < 4.0 IU/gHb. Unitized high and low G6PD controls are provided by the manufacturer to support quality assurance.

All microscopy confirmed *P. vivax* malaria patients were screened for G6PDd before PQ prescription in MTUs where the test was available. Each unit had its own device, but additional rotating devices were available for household screening before providing treatment. Chloroquine for three days plus daily (0.5 mg/kg/day for seven days) PQ or weekly (0.75mg/kg/week for eight weeks) PQ were used for G6PD normal and deficient patients, respectively, as per national guidelines [16]. Patients received a green or a red G6PD result card for normal and deficient results, respectively. The malaria card proposal was created to show the G6PD result and record the patient’s treatment schedule. HCPs were trained to advise all patients to return on the fifth day of treatment to monitor signs and symptoms of AHA, especially dark urine and jaundice [5]. If present, patients were referred to the nearest reference hospital. All malaria and G6PD data were entered in the national malaria notification system (SIVEP-malaria) by each municipality team.

### 2.5. Genotyping of A Subset of Samples

Patients with G6PDd results and approximately twice the number of randomly chosen normal results had samples collected on a separate visit to be used for genotyping. Blood samples were collected on filter papers, and were genotyped for the three most common G6PD variants (African A-, African A+, and Mediterranean) in the Brazilian Amazon [17]. Briefly, DNA was obtained by extraction using QIAmp DNA Mini kit (Qiagen, Hilden, Germany) following the manufacturer’s instructions. The A376G (rs1050829), G202A (rs1050828), and C563T (rs5030868) mutations were genotyped by TaqMan™ (Life Technologies, Carlsbad, CA, USA) assays using Applied Biosystems 7500 Fast real-time polymerase chain reaction (qPCR). Fluorescence curves were analyzed with the 7500 FAST Sequence Detection Software version 2.1 (Applied Biosystems, Foster City, CA, USA) for allelic discrimination.

### 2.6. Quality Assurance and Oversight

Study field supervisors oversaw all procedures, working closely with each malaria department supervision team. They performed quality control analysis with test instruments following each municipality supervision chronogram (2–4 weeks) using both levels provided by the manufacturer (Level 1: 0–3 IU/gHb and level 2: 6–17 IU/gHb). Tests were stored as per the manufacturer’s instructions at their respective Municipality Malaria Department for centralized distribution.

### 2.7. Perceptions of HCPs and Patients about Implementation

A purposeful sample of patients and HCPs participated in qualitative research according to the theoretical saturation criterion, by which focus groups discussions (FGDs) and in-depth interviews (IDIs) were performed until a clear pattern appeared and subsequent groups did not produce new information [18]. The goal was to understand in-depth perceptions about the treatment and experience with the quantitative G6PD test implementation. 

### 2.8. Data Collection

IDIs and FGDs were conducted pre-training and six months post-training with HCPs and six and 12 months post-implementation with selected (normal and deficient) patients from all malaria posts. HCPs were assessed regarding information on professional experience, education, knowledge on *P. vivax* malaria treatment, PQ use, acceptability of training and use of rapid tests in the field, G6PDd reporting, and antimalarial side effects, as well as recognizing knowledge gaps and suggestions for improving implementation strategies. 

A semi-structured interview guide with open-ended questions, complementary questions, and instructions was developed and previously validated in a small sample of participants, which allowed the interviewer to investigate the subject in more detail. Research areas were refined based on discussions and agreements within the interdisciplinary research team with experience in qualitative research and malaria. To assess user satisfaction with the introduction of the test into routine care, a sample of patients was selected to participate in IDIs. An interview guide was also prepared with questions about perception of pre-test orientation, test application and post-counseling, perceived utility, and satisfaction with the test application. The IDIs were carried out in quiet rooms at the participants’ workplaces and/or homes in the presence of a team observer who took field notes. The interviews lasted approximately 40 min, while the FGDs lasted approximately 60 min. All qualitative data were collected and analyzed by five authors with experience and training in qualitative research. 

### 2.9. Qualitative Data Analysis

The recordings of the interviews were transcribed and inserted into the MAXQDA20 program. Qualitative analysis was carried out through thematic framework analysis, and categories were created based on reading of the interview transcripts. These categories emerged during the analysis process and were discussed among the researchers for consensus. Two researchers developed a codebook and started line-by-line coding. In addition to recording IDIs and FGDs, researchers used data triangulation with field notes. The description of the qualitative methods in this article was crossed with the consolidated criteria for reporting qualitative research (COREQ) [19] (Supplementary file S2).

### 2.10. Statistical Analysis

Descriptive statistics were used for demographic, laboratory, and training data. Qualitative analysis was carried out as previously described [9,20].

### 2.11. Ethical Clearance

This protocol was approved by the Ethics Review Board at the *Fundação de Medicina Tropical Dr Heitor Vieira Dourado* in Manaus, Brazil (identifier CAAE: 92012818.1.0000.0005). HCPs and patients (and legal representatives, where applicable) gave approval and signed informed consent forms for the qualitative interviews.

## 3. Results

### 3.1. Study Population, G6PD Distribution and Treatment

**Figure 2:**
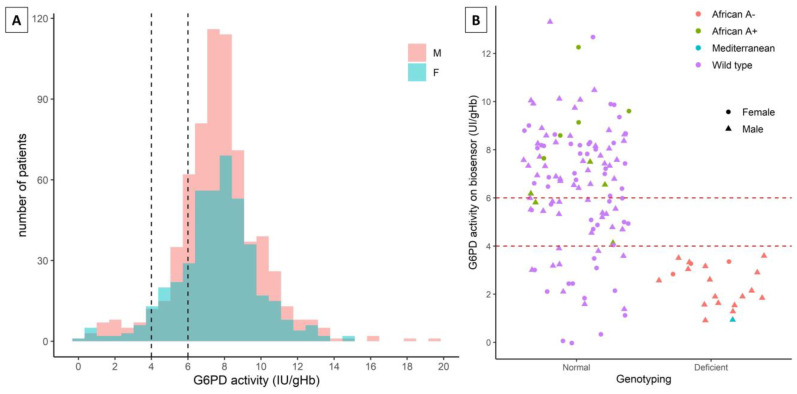
**G6PD activity in overall population and genotypes in a subset of 140 normal and deficient samples.** G6PD activity measured by the Standard^TM^ G6PD test in malaria patients is shown for males (M) in orange and females (F) in green (**A**). G6PD variants of 96 normal and 44 deficient genotyped samples are shown (**B**). G6PD African A-, African A+, Mediterranean, and wild type (non-African and non-Mediterranean) variants are indicated by red, green, blue, and purple colors, respectively. Circles below 4.0 IU/g Hb are considered G6PD deficient and between 4.0 and 6.0 are considered intermediate results (for females).

A total of 1654 *P. vivax* malaria patients were recruited with 656 being female (39.6%). Of these, 66 patients received a G6PD deficient test result across Rio Preto da Eva (January to August 2020) and Mâncio Lima (January to December 2020) with an overall prevalence of 3.9%. The study population is summarized in Table 1. The overall distribution of G6PD test results segregated by males and females is shown in Figure 2. 

Based on the treatment algorithm reported on the malaria form, 1230 study participants received 7-day primaquine and 56 received the 8-week once weekly primaquine course. Two male participants reported signs and symptoms of hemolysis, such as dark urine and jaundice; one had a normal G6PD result while the other had no information on G6PD activity in the malaria form.

### 3.2. Confirmatory G6PD Genotyping

Dried blood spots from a subset of 140 (81 males (M) and 59 females (F)) study participants from both municipalities were collected for confirmatory genotyping (Figure 2). The subset included all study participants with a deficient result (*n* = 44: 28 M, 16 F), and 96 (53 M, 43 F) randomly chosen participants with a normal result.

From all deficient results, only 21 (47.7%) had genotyping confirmation (20 G6PD African A- and 1 G6PD Mediterranean) as 18 hemizygous males and 3 heterozygous females. No G6PD deficient variants were observed among those classified by the STANDARD^TM^ G6PD test as either intermediate or normal. (Table 2).

### 3.3. Training and Reliability of Interpretation

In total, 101 health care professionals selected by the Malaria Departments were trained. After a single 4-hour theory and practical training session, most HCPs (73.4%) achieved the required passing grade (≤1 missed question or ≥90%) in both examinations. The most frequently missed step (>50%) during practice was confirming if the chip code number inserted in the instrument was the same as the one shown in the strip envelope. Results from the evaluations at 6 and 12 months after implementation varied among municipalities; in Mâncio Lima, minimum scores for both examinations remained steady (78.5% and 84.6% of participants at months 6 and 12, respectively) while Rio Preto had the lowest scores at month 6 (40.7%). 

Several trainings were carried out with the HCPs as mentioned above. However, a lack of adherence to the content by some professionals was noticed:

*“The training was very good, we practiced too. What stuck most in my mind was the treatment, because many people were in doubt about which medication would be used if the person could not take the medications daily. There were many people with doubts when the G6PD value was below four…”*

*(HCP001_081720)*

### 3.4. Perceptions about Test Usage

Despite the extensive and repeated training, some HCPs felt implementation of the G6PD test could be challenging. Challenges ranged from the reason for performing the test, to the associated workload increase, to test handling. Not all professionals who attended the training had an opportunity to keep using it in their routine practice, resulting in them forgetting the instructions after a certain period.

*“I don’t understand why this test is done. It was exactly with this doubt that I left the training…”*

*(HCP014_082020)*

*“On the day of training I had no difficulty handling it, but I’ll be honest, if you asked me to do it now I wouldn’t know how, because I did the training but I didn’t practice in the field. So for me it’s difficult.”*

*(HCP001_081720)*

*“The test is for finding people who have a reaction with chloroquine, not primaquine.”*

*(HCP012_082020)*

### 3.5. Malaria Card and G6PD Result Recording

A malaria card including G6PD data was given to patients with a malaria-positive thick blood smear. Among 1106 notification forms with a “source of G6PD information” field filled out, there were 61 occasions when the G6PD value on the card was used to inform treatment when the patient presented again with malaria. However, other uses were attributed to the card, both by patients and health professionals. The card was seen as a document that showed the patient’s history of malaria, to know when and what type of malaria he/she had within a certain period, while the G6PD result section received little or no attention.

*“It’s important because it’s like proof that I had malaria, I can get to someone and show that I had malaria, it’s like a document I think. That’s why I consider it important because even for me to travel, if I arrive at a place and there is a police barrier, I can show that I already had (malaria) and had the treatment and I can travel. My card is kept in my wallet, I always carry it, when I travel I like to carry my documents. I think it’s necessary to always show the card to prove that it has already happened. If I lose my card, it’s difficult for me to prove that I had malaria.”*

*(patient09_082020)*

### 3.6. Perceptions about Acute Hemolytic Anemia

Many HCPs were unable to associate the cause of AHA cases with the use of medication, believing instead that the signs and symptoms presented were caused by malaria or the lack of nutrients in the patient’s diet:

*“Anemia is when the person has weakness in the legs, lack of blood. That’s anemia. I don’t think it’s related to primaquine… but I can’t explain why not.”*

*(HCP014_082020)*

*“Hemolytic anemia, I think it’s a serious anemia, I think it’s due to having several malarias, and not because of the medicine.”*

*(HCP016_082020)*

Despite this, the HCPs were able to identify, through training, the signs and symptoms of AHA and their meaning, referring patients for medical treatment.

*“With the first doses patients always feel very ill, with dizziness, weakness, fainting. I’ve seen reports of yellowish eyes, dark urine, but that was with the first three doses of the medicine, they got better afterwards.”*

*(HCP013_082020)*

Some patients interviewed reported feeling more comfortable with the new treatment regimen (weekly PQ) to relieve the discomfort caused by the previous daily regimen. However, many health professionals believed that this represented a very small portion of the population, as most other patients were not comfortable with the new weekly treatment regimen and complained to HCPs about the long treatment time, the possibility of missed doses, and possible ineffectiveness of the drug.

*“In the case of patients who use the weekly treatment, they always report that they understand now why the urine was dark before, but now there are some patients who do not accept it, they say it will not cure, they think it is too much medicine and it has already happened that they abandoned treatment.”*

*(HCP010_081820)*

*“Patients prefer the seven-day treatment, especially those who like alcoholic drinks. Sometimes, mothers of young children don’t like the weekly treatment.”*

*(FGD001_081720)*

## 4. Discussion 

The quantitative G6PD screening performed by HCPs in MTUs showed good laboratory performance in real-world conditions. Results from validation studies have supported its use to mitigate limitations seen in qualitative testing platforms [11,15], but questions remained about how to incorporate it into routine malaria diagnostic units. An earlier phase of this study showed the ability of HCPs to perform the CareStart^TM^ qualitative test, which had an equivalent sensitivity to, but lower specificity than, the Standard^TM^ G6PD biosensor [9]. Data from controlled settings showed great diagnostic performance of the latter in reference units in Brazil [15] and good repeatability and inter-laboratory reproducibility were observed in different laboratories [21]. When the test is used in less controlled environments, the performance can be affected by external factors. Increased G6PD activity can also be observed during acute malaria infection [22]. Nonetheless, the use of the test in real-life settings was shown to be cost-effective in avoiding hospitalizations [23].

The prevalence of G6PDd cases in this study population as determined by the Standard^TM^ G6PD test was similar to the 4.5% reported in previous studies [6,24]. Despite this, genotyping most samples with less than 4.0 IU/g Hb G6PD activity only identified G6PDd genotypes in 21/44 cases. As previously shown, no cases in the study with G6PD activity greater than 4.0 IU/g Hb had a G6PDd genotype [15]. So while the genotyping supported a 100% negative predictive power for G6PD deficient cases in *P. vivax* infected individuals with G6PD activity greater than 4.0 IU/g Hb, the test did show a slightly lower positive predictive power among those that are classified as G6PDd by the Standard^TM^ G6PD test compared to the clinical evaluation [15]. Operationally, this means the test can still inform safe treatment of patients with primaquine, and the strong negative predictive power suggests that those classified as eligible for primaquine (1230/1286) do not need re-testing if G6PD testing records could be kept. In contrast, those classified as G6PDd by the Standard test would ideally receive confirmatory testing. Operational studies are required to investigate if sequential testing on the Standard^TM^ G6PD could improve the positive predictive power. This false positive rate represents individuals who could otherwise receive primaquine. Data from the same municipalities show that the use of weekly PQ did not increase recurrence rates compared to the standard of care [25]. Further studies are also required to better understand the decrease in the observed positive predictive power between the clinical evaluation study reported previously and the operational study reported here.

A different set of skills are necessary to perform the quantitative Standard^TM^ G6PD test, which also includes two quality control procedures. Previous data from Brazil showed HCPs were able to understand its use and importance after minimal training [26]. Although most professionals were able to correctly perform the test after training, knowledge consolidation can be achieved with practical use as observed in scores between M6 and M12 (higher scores in Mâncio Lima compared to Rio Preto da Eva). Concerns over technical aspects of the test were observed, especially in units that diagnose few malaria cases annually. Such aspects can affect test performance due to operational errors. Earlier studies in different populations also report similar perceptions from HCPs about the same test (e.g., insufficient mixing of samples, bubbles in the sample collector provided, etc.) [27]. In units where malaria cases are not frequent (e.g., Rio Preto da Eva) refresher training sessions are advised, to mitigate technical errors such as the ones mentioned above, at least every 6 months.

The perception of hemolysis not associated with treatment with PQ in G6PD deficient patients was frequent in interviewed HCPs. HCPs have been diagnosing and treating *P. vivax* malaria for decades, regardless of the patient’s G6PD status. Therefore, even after training was performed and considered successful by the HCPs, gaps in knowledge about G6PD were observed. Lack of awareness of the importance of testing prior to the use of PQ to prevent hemolysis can be a major barrier to definitive implementation of the G6PD test. A similar barrier was observed in some interviews carried out with HCPs and policy makers from Bangladesh, Cambodia, China, and Malaysia [28]. Thus, in addition to training, it is important to provide educational materials based on local perceptions/culture that promote effective communication between HCPs, patients, and policy makers.

Another barrier to the implementation of the G6PD test presented by the HCPs was the increase in the workload at the beginning of the study, especially with the Biosensor. Similar complaints were reported in other countries [27]. However, in our study some HCPs showed a different perception in interviews six months post-training, appreciating the benefits of the test in reducing patients who had hemolysis. Therefore, demystifying the increased workload of HCPs should be considered in training/educational materials in areas that will implement the G6PD test. The main bottlenecks and their possible countermeasures are summarized in Supplementary file S3.

This study had limitations. Directly supervised therapy (DOT), although recommended for weekly PQ, could not be ensured due to the pragmatic approach of the study. The reference assay for G6PD genotyping detected only the most common circulating variants; therefore, additional variants might not have been detected. Further investigation, such as gene sequencing, is advised for G6PDd test cases with no variant found by genotyping. Although there is no standardized timeline for quality control recommended by the manufacturer, all devices showed good performance and a high rate of success using quality controls without any apparent technical malfunction noticed during implementation. Findings from the qualitative assessments might not be generalizable to other settings. As this study adopted a qualitative approach with intentional non-probabilistic sampling, results do not necessarily represent the perceptions of all patients and health professionals in the Brazilian Amazon. Despite efforts to minimize interference, the interviews may contain some, inherent to a qualitative approach, especially when reporting the behavior of other people in the community.

In conclusion, the Standard^TM^ G6PD quantitative screening test performed well in reducing the possibility of exposure of G6PD deficient patients to high dose primaquine in real-life settings of the Amazon and showed good acceptability by health care professionals and patients. Further measures are recommended to address technical limitations and perceptions observed here to expand G6PD screening to larger settings.

## Figures and Tables

**Table 1:** Study population data.

Variable	Rio Preto da Eva *n* = 413	Mâncio Lima *n* = 1241
Females, *n* (%)	159/413 (38.5%)	497/1241 (40.0%)
Males, *n* (%)	254/413 (61.5%)	744/1241 (60.0%)
Median age (SD)	32.3 (18.7%)	24.6 (17.2%)
**G6PD Activity Ranges**		
<4.0 IU/gHb (M/F)	15/8	24/16
≥4.0 and ≤6.0 IU/gHb (M/F)	16/10	63/59
>6.0 (M/F)	56/36	505/298
**Radical Cure Treatments**		
Study participants prescribed 7-day primaquine	126/150 (84.0%)	1104/1208 (91.4%)
Study participants prescribed weekly dose primaquine	22/150 (14.7%)	34/1208 (2.8%)
Other	2/150 (1.3%)	70/1208 (5.8%)

Differences in totals are influenced by completeness of data reported.

**Table 2:** Distribution of G6PD genotypes across G6PD activity ranges as defined by the STANDARD^TM^ G6PD test.

	**Males (*n* = 81)**		**Females (*n* = 59)**		
**STANDARD^TM^ G6PD** **Test Result**	**Hemizygous** **Deficient**	**Hemizygous Normal**	**Homozygous** **Deficient**	**Heterozygous**	**Homozygous** **Normal**
<4.0 U/gHb	18	10	-	3	13
≥4.0 and ≤6.0 U/gHb	-	15	-	-	8
>6.0 U/gHb	-	38	-	-	35

Genotyping description only for African A- and Mediterranean variants.

## Data Availability

De-identified data can be obtained from the corresponding author upon formal request.

## Supplementary Materials

The following supporting information can be downloaded at https://www.mdpi.com/article/10.3390/pathogens11111328/s1, Supplementary File S1: Theory and practice examinations; Supplementary File S2: Consolidated criteria for reporting qualitative research (COREQ); Supplementary File S3: Bottlenecks and countermeasures of implementation.
