# Supplementary material for: Quantitative G6PD Deficiency Screening in Routine Malaria Diagnostic Units in the Brazilian Amazon (SAFEPRIM): An Operational Mixed-Methods Study
Source: Pathogens. 2022 Nov 11;11(11):1328. doi: 10.3390/pathogens11111328 (PMC9696723; doi:10.3390/pathogens11111328)
Supplement: Supplementary file 1 [file pathogens-11-01328-s001.zip › Supplementary File S1.pdf]

THEORY TEST

HCP NAME:____________________________________HCP SIVEP CODE:________
HEALTH POST: _________________________________ DATE _____/_____/______

Choose ONLY ONE alternative for each question:

1. When should the test be performed? Após que malária o teste deve ser feito?

a) vivax and mixed malaria (Pv and Pf)
b) falciparum malaria
c) any malaria

2. Why is primaquine taken for?

a) To treat liver-stage forms (latent forms in the liver) that lead to a new malaria episode even without a mosquito bite
b) to treat blood-stage forms, seen under microscopy, that are responsible for fever and chills
c) to treat headache

3. Which treatment is best for a patient with vivax malaria and primaquine test result of 2.0?

a) chloroquine – 3 days and primaquine – 7 days
b) chloroquine – 3 days and weekly chloroquine for 12 weeks
c) chloroquine – 3 days and weekly primaquine for 8 weeks

4. Which treatment is correct for a patient with vivax malaria and primaquine test result of 11.2?

a) chloroquine – 3 days and primaquine – 7 days
b) chloroquine – 3 days and weekly chloroquine for 12 weeks
c) chloroquine – 3 days and weekly primaquine for 8 weeks

5. Which alternative shows what must be informed to each patient after primaquine test result and receive treatment?

a) monitor signs and symptoms of hemolysis, including coca-cola colored urine, yellow eyes and dizziness during treatment
b) return for verification blood smear on D5 of treatment
c) all options above

6. The following error message appeared on the biosensor display during test procedure. What does it mean?

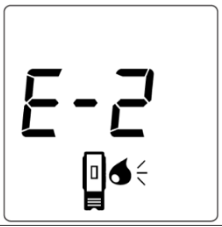

a) insufficient sample
b) temperature outside operating range
c) low battery

7. What must be done if a patient returns to health post presenting coca-cola colored urine and yellow skin?

a) refer to specialized health center (FMT-HVD)
b) repeat the primaquine test
c) perform a new thick blood smear

8. If a patient has a primaquine test result of 2.0:

a) He can use daily primaquine for 7 days
b) He has no malaria
c) He cannot use daily primaquine (will use weekly primaquine instead)

9. What may happen if a patient present coca-cola colored urine, yellow eyes and skin, tiredness and dizziness during treatment and he continues taking daily primaquine?

a) nothing
b) he may present severe anemia, requiring hospitalization
c) he will have another malaria

10. Identify the letters

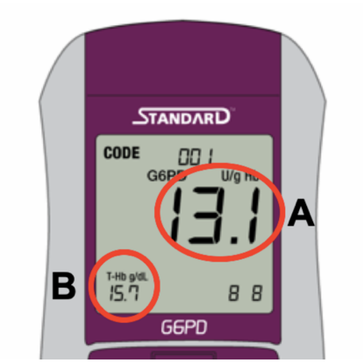

a) A – Test result (G6PD) / B – hemoglobin
b) A – hemoglobin / B – teste result (G6PD)
c) A – date / B – chip code

PRIMAQUINE TEST EVALUATION CHECKLIST

Participant's full name: ____

Agent's SIVEP Code: ______________________ Function: ______________________

Health Facility: ____

Date: ______________________ Instructor: ______________________

<u>Instructor's guidance for the standardization of the practical presentation</u>

<u>Instructor, ask the participants to gather in groups of 5 health care professionals (HCP). They should practice the blood collection among themselves, in case control kits are not available at the time of practice. Every HCP must perform at least ONE test CORRECTLY before proceeding for theory-practice evaluations. Ideally, it must take, each agent, a maximum of 5 minutes to perform the test. Common procedures such as filling out the SIVEP form and counseling the patient before and after the test should be performed together.</u>

Steps:

1) Start with how to explain to patient the use of the test:
   a) *It is to verify the best primaquine scheme for the patient*
   b) *Some patients may develop reactions to daily primaquine (mention coca-cola colored urine and yellow skin/eyes) that may lead to hospitalization.*

2) Perform the first test, explaining <u>IN DETAILS</u> each step, from the codechip and expiry date verification, specimen collections, procedure up to result interpretation and counseling. Make sure to show all necessary materials and procedures, including the checkstrip procedure and its use (if the SDB instrument falls, when opening a new test device kit, after changing batteries etc). Make sure they're collecting blood up to the black line, explaining how to hold the sample collector to collect and release the sample.

3) Ask participants to start supervised practice, using a coworker as a patient.

4) Make comments on the most common errors and what to do while waiting for the test result.
   a) *At least E-1 error (test device inserted improperly or damaged), E-2 (insufficient amount of blood specimen) and E-4 (temperature above or below the operating temperature range) and what to do.*

5) After the results of 1 or 2 participants, show how to fill out the test results, D5 visit, and treatment on the SIVEP form. You don't have to repeat it more than twice.

6) Similarly, comment on how to instruct the patient to return to D5 visit, monitor symptoms of hemolysis and loof for a specialized medical center.

7) After all of them have performed at least one practice round, proceed to theory evaluation. Refer them to one of available practice evaluation stations as they finish.

8) Only the test procedure and filling of the SIVEP form must be officially graded (items 2.2 to 4.2, one point each) for certification. Minimum approval grade is 90%. Comment and reinforce other non-graded topics as HCPs fail to perform correctly.

Instructor, please, guide the health care professionals about what will be evaluated (don't forget to mention each main topics from 1 to 5) and what the agent is supposed to do, before he/she begins the practical test. Only intervene or comment by the end of this section, observing the deficient points during the practical procedure.

1. <u>About informing the patient:</u>

1.1.	Did the agent talk about the purpose of the test and its importance? *The evaluator can directly ask "What is this test for?"*	**YES ( )**<br>**NO ( )**

2. About the organization of required materials:

2.1.	Did the participant organize all the materials required for the test (one test device, two sample collectors, one extraction buffer, lancet, gloves, cotton balls)?	**YES ( )**<br>**NO ( )**
2.2.	Did the participant check if the chip code match the number printed on the foil pouch?	**YES ( )**<br>**NO ( )**

3. About performing the G6PD test:

3.1.	Did the participant discard the first drop of the patient's blood?	**YES ( )**<br>**NO ( )**
3.2.	Did the participant collect the blood specimen up to the black line?	**YES ( )**<br>**NO ( )**
3.3.	Did the participant mix the collected specimen with the extraction buffer pressing and releasing the sample collector 8 to 12 times?	**YES ( )**<br>**NO ( )**
3.4.	With a new sample collector, did the participant collect the mixed specimen to up to black line?	**YES ( )**<br>**NO ( )**
3.5.	Did the participant apply the mixed specimen to the specimen application hole of the test device and immediately close the flap?	**YES ( )**<br>**NO ( )**
3.6.	If there was an error message, did the participant know how to proceed with? *Choose not applicable (N/A) if there was none.*	**YES ( )**<br>**NO ( )**<br>**N/A ( )**

4. About filling out the SIVEP's form (use the form available in the participant's kit):

4.1.	Did the participant fill out the field for test and hemoglobin results correctly (48.1 e 48.2)? *Help the agent to identify the correct field, if needed.*	**YES ( )**<br>**NO ( )**
4.2.	Did the participant choose the correct treatment (46.1 e 46.2)? *Help the agent to identify the correct field, if needed.*	**YES ( )**<br>**NO ( )**

5. About counseling the patient for signs and symptoms of hemolysis:

5.1.	Did the participant talk about returning on the 5th day of treatment (LVC D5)?	**YES ( )**<br>**NO ( )**
5.2.	Did the participant talk about the monitoring of signs and symptoms of hemolysis? *Mention at least coca-cola urine and/or yellow eyes and skin.*	**YES ( )**<br>**NO ( )**
5.3.	Did the participant talk about the importance of looking for specialized medical center if signs or symptoms of hemolysis are observed?	**YES ( )**<br>**NO ( )**
