# Supplementary figures and images for: Quantitative G6PD Deficiency Screening in Routine Malaria Diagnostic Units in the Brazilian Amazon (SAFEPRIM): An Operational Mixed-Methods Study
Source: Pathogens. 2022 Nov 11;11(11):1328. doi: 10.3390/pathogens11111328 (PMC9696723; doi:10.3390/pathogens11111328)

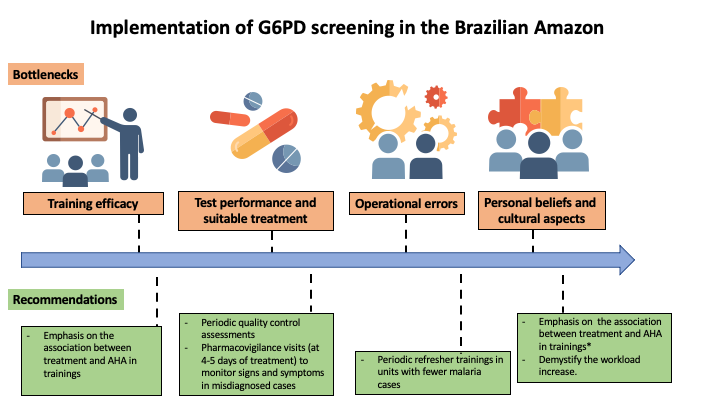

Supplement: Supplementary file 1 [file pathogens-11-01328-s001.zip › Supplementary File S3.tiff]
